# Treatment of Dental Caries, Etc.

**Published:** 1851-10

**Authors:** Robert Arthur


### Selections.

*Treatment of Dental Caries, complicated with Disorders of the Pulp and Peridental Membrane.* By ROBERT ARTHUR, D. D. S.

I PROPOSE now to consider those cases in which the caries has passed that point when it can be removed, in the performance of the necessary operations to arrest its progress without danger of injury to the pulp; or after it may have begun to exercise some injurious influence upon this part of the affected tooth.

The principal portion of these papers will be devoted to a discussion of the propriety, in certain cases, of the entire extirpation of the dental pulp, and the best means of effecting this object. And although I shall endeavor to show that this practice, in certain cases, may be pursued with great advantage, I am quite willing to admit, what, indeed, I could not deny, that when the pulp can be preserved *untouched, in a healthy condition*, it is always better that it should be done. No effort, indeed, should be spared to accomplish this most desirable object.

In treating this part of my subject, I shall present views at variance with those generally entertained in relation to it, in the profession; views, however, the correctness of which can, I think, be clearly established, both by sound reasoning, and by facts. Although I am not aware of any publication which will bear me out in the position I am about to take, I am gratified to know, from private intercourse with some of the most intelligent members of the dental profession, that my opinions in this regard are not peculiar, and that if I am not sustained by them to the full extent to which I propose to carry them into practice, still I am so to an extent far beyond what has ever been sanctioned by any writer upon dental surgery, at present known to me.

I deny, positively, the truth of the statement so often made, that caries of the teeth will always continue to progress, after it has once set in, unless every particle of the decayed bone be removed from contact with that which is healthy. I do not believe that, when the cavity of decay is so filled, as absolutely to prevent ingress of the fluids of the mouth, that the disease ever advances; although my observations upon this point are not sufficient to furnish me with decisive proof that it is so. But I do affirm, with confidence, that a series of observations, running through a practice of ten years, have convinced me that in a vast majority of cases, caries of the teeth remains stationary, if the opening of the cavity of decay is well prepared and the cavity so filled as to exclude every thing from it, although decayed, dead and decomposed bone be left in it.

This opinion is not advanced in a spirit of self-confidence, or for the vain purpose of endeavoring to gain reputation by presenting a novelty. I state it, and shall endeavor to establish its truth, because, as I shall show, if grounded in fact, it must have a most important practical bearing.

What is the true pathology of dental caries?

After a great deal of discussion of this question, extending over a number of years, the settled opinion at the present day is, that caries or decay of the human teeth, is a decomposition of the dental substance, caused by the action of a chemical agent formed, in most cases, in the mouth, and brought into, and retained in contact with certain parts of the teeth; its action modified by a variety of circumstances, more or less perfectly understood. I believe that this view of the nature and cause of dental caries is not now controverted.

How, indeed, can it successfully be? It is known, to demonstration, that certain kinds of acids are capable of decomposing the earthy bases entering into the structure and forming a large part of the dentine and enamel of the teeth. It has been repeatedly demonstrated that such an acid is present in the mouth of many, if not of most persons. And it is known that in cases where decay has begun, and would inevitably go on—as, for instance, on the proximate surfaces of the teeth—if such a change is made in them as to prevent the continued retention of the destructive agent, decay will cease to advance.

It is then, a fact, so well established by long and general observation, that I think I may take it for granted, that the exciting cause of decay of the teeth is external, and that the destructive agent, whatever it may be, is in the mouth, and exerts its influence upon the teeth, by being retained in contact with certain parts of them. Its action is, of course, modified by the original structure of the teeth.

If this be admitted, the only question having a bearing on this discussion is: Do any new elements enter into this destructive process, after it once begins, rendering its progress independent of the original cause?

I have examined, carefully, all the works on dental surgery within my reach, but find nothing in them to assist me in this inquiry. This, I believe, has never been made a subject of special investigation, and all the inferences drawn from the presentation of the general subject of caries of the teeth, point in an opposite direction.

In an extensive intercourse with intelligent members of the profession, I have frequently introduced this subject, but have not yet heard a distinct suggestion at all satisfactory to me, as to the manner in which caries of the teeth could progress, except by the action of some external solvent.

The only way, within my knowledge, that it has been attempted to be accounted for, with any show of reason, is upon the old hypothesis of inflammation of the dentine. Now, it has many times been most conclusively shown, that in the commencement, at least, inflammation of the dentine is not the cause of caries. And even, if it may be supposed that decay is kept up, after having once set in, from this cause, in what manner could carious bone contribute to this result? I am not aware, that decayed dentine has has been supposed, by any writer worthy of consideration, to contain any particularly corrosive qualities; and, if it is only inorganic matter, which it must be—simply, dental bone deprived of its vitality—I am unable to conceive of the manner in which this could serve to keep up the inflammation of the part with which it is in contact any more than the materials used for filling, which are equally inorganic. It may be objected, that the carious bone will contain some of the solvent agent in union with it, and that this is sufficient to promulgate the disease. But, it must be seen that unless a further supply were furnished, this would soon exhaust itself and become neutralized.

If it were true, that inflammation of the dentine is a sufficient cause for the continuance of its decay, then the only means of arresting it of which we know anything, would prove worse than useless, for the direct contact with the living bone of a metallic substance, being, as it is, so good a conductor, would tend to keep up irritation instead of allaying it.

But, even if it be granted, which I do not grant, for facts show conclusively that it cannot be so, that decayed bone left in the cavity of decay could excite inflammation of the adjacent bone, I cannot conceive how this would bring about a condition of things bearing any resemblance to what occurs in the progress of dental caries. It might possibly cause death of the part; but, if after that, its decomposition could occur, I have observed no fact which would justify me in the conclusion, that the absorbent vessels of the tooth have sufficient capacity to remove the decomposed bone.

Mr. Tomes, in his account of the causes of dental caries, supposes it necessary that the dentine should first be deprived of its vitality before it can be acted upon by the decomposing agent. But, although he makes the death of the part an essential preparation for the action of the solvent upon it, he makes the decomposition of the dental tissue absolutely and invariably dependant upon a solvent agent. How far he may be correct in his position, in relation to the deprivation of the dentine of vitality previous to the occurrence of decay, I will not now undertake to examine. It is probable, that destruction of vitality does take place before decomposition, but the agent causing the decomposition of the part is, in itself, capable of destroying its vitality, and, in my opinion, generally does so. Mr. Tomes, however, attributes this loss of vitality, in many instances, to constitutional causes acting independently of the agent producing the caries, and preparing the way for it.

A great deal has been said about the removal of the dead and decomposing dentine, in performing the operation of filling, and the necessity of cutting down to that which is sound and healthy, so as to allow no contact between the dead and living bone. But, it must not be forgotten, that the surface of the bone which is cut away, no matter how sound and healthy it may be at the time of the operation, cannot be treated in this manner without injury. Such violence as cutting across a living part cannot be done without injury to its vitality, and if injured, we know that the dentine has no power of recovering itself, and, at last, it must be seen that the surface of the cavity next the filling is dead bone, or bone partially deprived of its vitality.

If this be true, it is evident that the simple death of a part of the bone cannot produce decay in an adjacent part; for if it were so, the operation invariably resorted to for the arrest of this disease, instead of being perfectly effectual for the purpose, as it is found to be, would be the very means of propagating it more rapidly, for the death, or partial death, which amounts to the same, of a considerable part of the bone, is the invariable result of the preparation for filling of every cavity.

Judging, therefore, from a consideration of the structure of the teeth, the low degree of their vitality, the only means of which we know anything by which their decomposition is effected, I cannot but conclude that dental caries, as commonly known, is invariably caused by the action of some solvent agent, retained long enough in contact with the bone to effect its decomposition, and that if the contact of this destructive agent can be effectually prevented, the decay cannot go on.

I am most decidedly confirmed in this opinion, by all the facts having any bearing upon the matter, which have come within my practice for a number of years. Many of these instances must have been met with by every one engaged in the practice of the profession.

A fact well-known, although trifling in itself, goes a great way to establish the position I have taken; indeed, I am unable to see how such evidence can be set aside. I allude to the fact, that if a deposit of tartar takes place in a cavity of decay, the destructive process is most certainly arrested. The late Dr. Hayden, I well remember, was in the habit of saying, that if he found a decayed cavity filled with tartar, he never touched it, for the preservation of the tooth in that condition was quite certain. Now, in this case, nothing has been done toward the removal of the carious bone, upon which the tartar has been deposited—the cavity is in precisely similar condition to one in which a gold filling has been placed upon the carious bone. The objection may be made here, that the character of the saliva, from which the tartar has been deposited, is itself a sufficient reason to account for the arrest of the decay; for the very deposit is an evidence of an excess of alkaline qualities, and it must have neutralized any acid secretion present, and prevented its action upon the bone previous to its deposit. But, this objection only goes farther to establish my view of the case, for it would prove, that as soon as the action of the acid agent is interfered with the decay is arrested. It may also be said, that there is this difference in the two cases; that the gold placed upon the decayed bone, confines in the cavity a portion of the destructive agent, which is completely neutralized before the deposit of tartar commences. But, it is only necessary again to reply, that the quantity of acid which could in such case be left in union with the decayed bone would be so trifling that as soon as a further supply was cut off it would speedily exhaust itself.

I have conversed on the subject with but few of the better practitioners of our profession, who have not admitted, that, occasionally, they regard it as good practice to leave decomposed bone in the bottom of a cavity when it was very probable, that the removal of this changed bone would lay bare the pulp. By decomposed bone I do not now mean that discolored bone which is so often thrown out by the pulp when threatened with exposure. That this practice is more commonly pursued than is generally acknowledged, I am fully satisfied. In these cases, the caries must have penetrated so nearly to the pulp, that if it continued to progress with the same rapidity with which it had gone on to this point, a few weeks or at most months, would have been sufficient to have required the removal of the filling, pulp or the tooth, and yet I have not heard the individuals here alluded to, imply that they regarded this as an operation requiring subsequent attention.

I have removed fillings in consequence of their defectiveness, which have been in place for many years, and have repeatedly found what I could not but regard as conclusive evidence, that the caries had not been entirely removed at the time the operation was performed, and yet decay had not progressed in the parts where it might have been expected to go on. It seems to me scarcely possible that this should not have been the experience of every practitioner of long standing.

I have mentioned these things as facts, probably coming within the observation of every practicing dentist. But in addition to these, I have, for several years past, been making experiments with direct reference to this particular object. The results of these observations I will, at some future time, make known more specifically to the profession. These facts, to which I have endeavored to direct attention, although they may not lead every practitioner so far in his conclusions, as they, in connexion with other facts have led me, are sufficient,

I think, to establish conclusively this point, which for the present is sufficient for my purpose; that if the cavity of decay is securely filled so as perfectly to exclude the fluids of the mouth, the caries will be materially arrested. I cannot see how, in the face of these well-known facts, this can be controverted.

It is now generally admitted, that after the human teeth have attained to their destined length, a deposition of bone from the pulp generally goes on, eventually, in many cases, bringing about its entire obliteration. It is well-known that this deposition of bone, from a variety of causes, goes on, at times, more rapidly than it ordinarily does, and sometimes when the pulp is threatened with exposure from decay, is thrown out in such quantity as entirely to arrest the progress of this disease. The reason why this does not always happen is, that the caries progresses so much more rapidly than this deposition of bone takes place, that the pulp becomes uncovered or brought into contact with the carious bone, and no longer performs this important function. There is no reason to believe that bone is not invariably thrown out from the pulp, and if, when this important part of the tooth is on the point of becoming uncovered, the progress of the caries can be retarded, it is no violent inference to suppose, tnat, in time, a thick plate of bone will be formed between it and the cavity of decay.

It is well-known to the profession, that a tooth may be decayed so nearly to the pulp, that the bone in direct contact with this part, may be in a soft condition without causing any apparent inflammation or even considerable irritation of the pulp. And even in cases where some irritation is present, a degree of inflammation so high as to cause pain does not occcr. There may even be great tenderness to slight pressure, so nearly may the pulp be entirely uncovered, and yet the pulp seem unaffected by disease.

I do not suppose, however, that any portion of the healthy pulp can remain directly in contact with devitalized bone, any more than with any other inorganic substance, without suffering the usual consequences. It seems probable to me, that in those cases where the bone no longer retains its proper consistence down to the surface of the pulp, and no painful symptoms are present, that a new layer of bone has been formed on the surface of the pulp, and has not yet become hard. It may readily be seen that in such a case as this, although healthy living bone may be in direct contact with the pulp, it would not have consistence enough to protect it from injury by external pressure. It is obvious, too, that in the attempt to remove the decayed parts near it, this new bone may be as easily removed as that which is decomposed. Pain is probably rarely experienced except when, by the caries proceeding more rapidly than this protective deposition of bone, the pulp is at last brought into direct contact with the carious bone, or so large a part is left bare as to expose it to the influence of irritating substances taken into the mouth, or of the atmospheric air. This is a matter well worthy of consideration, and it must be remembered that when directions are given for the invariable removal of every particle of soft bone from a cavity of decay, that bone which has less consistence than the rest of the tooth, may be bone in the first stages of formation.

The principal reason why I am so anxious to establish the truth of the position I have taken, is, that after the pulp once becomes fully exposed, even the smallest point, the preservation of teeth so affected, is, in my estimation, very doubtful, unless the pulp is entirely removed. The treatment of a tooth in this condition, by "capping" the pulp, as it is termed, has been warmly advocated, but I must affirm, although my experience in the operation has been very limited, that I do not remember to have been completely successful in the treatment of a single case in this way. In conversations on this subject with skilful practitioners, many of them have stated this to have been their own experience.

Dr. Westcott, Journal, vol. vi, p. 112, in a strong article condemning the use of arsenic for destroying the vitality of the pulp, says of this operation, that he has "tried the experiment time and again, and has never succeeded in a single instance."

I have recently had conversations with members of the profession, who, at one time, very warmly advocated this practice, but who now express doubt as to the final result, and advise that it should be practiced with extreme caution.

I have, on the other hand, been assured by Professor Harris, who has practiced this operation very extensively, that he had removed fillings after some time had elapsed, and found the opening, into the pulp cavity, which was quite distinct at the time of the operation, completely closed with a deposit of new bone. Professor H. also states, that he has recently met with several well marked cases, in which the pulp was extensively exposed at the time of filling, but which, on removing the fillings for examination, was covered with bone in various stages of formation.

I am aware that this operation is practiced, and is strongly commended by other gentlemen of high standing in the profession, in whose statements I have confidence; and I am quite willing to confess, that I may not yet have fallen upon the correct method of practicing it. But I carefully performed the operation in the manner directed, and so long as I am unable to obtain any more favorable results, I cannot recommend the practice.

It may be asked, why not perform this operation, even if there is a slight prospect of success; for even if it fail, you can then resort to the extreme operation of removing the pulp? The objection is this: the preservation of the tooth is endangered by this course; for it will, in many cases, scarcely be discovered that the pulp is dead, until peridental inflammation occurs, and then the chances of the success of the final operation of the extirpation of the pulp are abridged; for as I shall endeavor to show, in the proper place, the success of this operation depends, to a considerable extent, upon the healthy condition of the pulp and the peridental membrane at the time it is performed.

It must be admitted, I think, from the evidence in relation to this matter, either that the true method of performing this operation is not generally understood, or that its success is very uncertain. I cannot but doubt the possibility of the pulp throwing out new bone after it is once entirely deprived of that with which it is naturally covered, it must be brought into contact with the atmosphere, and be more or less injuriously influenced by it. My own experience, in relation to this matter, confirms me in this view of the case.

I propose, now, basing this recommendation on the general view of this subject I have presented, that in every case occurring in practice, where there is a probability of exposing the pulp by the entire removal of the decayed bone to allow it to remain, even when necessary, in considerable quantity.

Let us now consider well this class of cases. It is admitted, on all hands, that no matter how carefully performed, there is some uncertainty, slight though it may be, about the final result of the operation, having in view the entire extirpation of the pulp. Although an invaluable operation in cases where it is absolutely demanded—and such cases present themselves quite often enough—it must be acknowledged to be a last resort The evidence in relation to the operation of capping the pulp, which I have just presented, shows, I think, that the successfull result of this mode of procedure is still more uncertain. Now, admit that we cannot calculate, with any degree of certainty, what will be the consequence of filling a cavity without entirely removing the decayed bone, is it not better, until we satisfy ourselves in relation to it, to make the trial than to go on so far as to oblige us either to perform the operation, difficult, out of the reach of the purses of some of your patients, and somewhat uncertain of removing the pulp, or the still more uncertain one of "capping the nerve." We know that a deposition of bone from the pulp is slowly but certainly going on; and we have good reason to believe, that if the progress of the caries can be stayed for a sufficient time, a hard and healthy covering to the pulp will have been supplied. I have been endeavoring to show that the operation I propose, if it do not entirely arrest, will certainly retard the progress of this disease; and, even if my treatment of the subject has not been convincing, a trial, at least, when I positively assert what has been my own experience in the matter, is more than justifiable when the object to be gained is of so much importance. We know, in many of these cases, where the removal of soft bone would certainly expose the pulp, that this bone remaining in contact with the delicate structure, produces no irritation, and from all the indications presented, leaves it in a perfectly healthy condition. Now, as I have already stated, it is the opinion of some of those who have extensively practiced this operation of "capping the nerve," even after it may have become exposed at a very small point, that its success is very uncertain. Others allege that they have failed in every case in which they have attempted it. Now, in view of this evidence in regard to it, is it not better to allow to remain in contact with the pulp a substance which seems to be perfectly congenial with it, and which produces no irritation, than to place in contact with it, a substance which experience shows is liable to produce the injurious results alluded to?

The following case is the best answer to these questions:

Case.—Mr. K——, æt. about forty-five. Had occasion to perform a number of operations upon this gentleman's teeth about two years ago. On the left side of the lower jaw the dens sapientæ alone remained. It had been decayed for a considerable time, and from its appearance I concluded that the pulp must be reached. It had given him no continued pain, however, but was exceedingly tender to pressure, so that it was quite useless for mastication. For several reasons, unnecessary to state here, I was unwilling, except as a last resort, to extirpate the pulp, and determined to make an effort to save it without resorting to this extreme measure. On examining, with an instrument, the cavity, which occupied nearly one-half the diameter of the surface of the crown, I found it extremely tender to the slightest touch, and could remove but a small portion even of the decayed bone, the greater part of which I allowed to remain in the cavity. Indeed, I did scarcely any thing more than to take away the very much softened portion. I then dried out the cavity as well as I could and filled it with tin foil. The tenderness under very slight pressure, however, was so great that I could not use force enough to make any thing like a good filling. For several weeks after this operation was perofrmed, the tooth remained as useless as ever, but it grew, eventually, less and less tender to pressure, and after the lapse of a few months it could be used for mastication without inconvenience. In about a year the tooth again became annoying; slight pain being experienced when certain condiments were taken into the mouth. On examination, I found that in consequence of the imperfect manner in which it had been filled, the caries had gone on at one side and formed a passage to the tender part of the cavity. I now took out the filling and found that the cavity had not increased at all in size, and I was enabled to remove the greater part of the discolored bone with but slight inconvenience to the patient until I came toward that point over one of the anterior elevations of the pulp. The rest of the cavity I prepared carefully, and again filled with tin foil so firmly, that at present, after the lapse of nearly eighteen months, it has been as useful as if it had never been touched with disease. In a few months I shall remove this tin filling, and fill firmly and finally with gold.

There is one objection to this practice; it may, like the operation of "capping" the pulp, endanger the final preservation of the tooth, if it become necessary to remove the pulp. For if the operation do not succeed perfectly, and the case is neglected, an inflammation of the peridental membrane may ensue, which will lessen the chances of the final preservation of the tooth under treatment. This difficulty may be avoided, as I think I shall be able to show, and the chances of success so greatly preponderate, and the advantages of this method of treatment are so considerable, that, in the face of this objection, I regard it as more than justifiable that this risk should be taken.

In the treatment of teeth in the condition here alluded to, there are many things requiring special attention. Most of these will doubtless suggest themselves to every practitioner of experience who can admit the correctness of the principle I

have suggested. It may, however, be useful to some to state in some detail my own general method of treating these cases.

The standard to which we must refer all preservative operations on the teeth, is undoubtedly this: what can we do in each individual case, to preserve the teeth for the longest period of time in the most healthy condition.

If the ground I have taken could be established beyond a doubt, then the matter would be quite easy, for we would not be solicitous, in any case, entirely to remove the decay from any cavity which came under treatment. But as this is a question, it is desirable in all cases to remove every trace of decay, and it becomes important to consider well the circumstances in which this may or may not be done without risk of injury to the pulp. Unless, of course, this practice is not approved of, and it is thought better to expose the pulp than to leave in the cavity of decay any quantity, no matter how small, of any thing like decomposed bone.

In examining a cavity of decay we may often at once satisfy ourselves of its proximity to the pulp, by considering the class to which the affected tooth belongs, the situation and extent of the decay, the condition of the cavity and the symptoms observed by the patient. At other times it is extremely difficult, without taking such steps as will render impossible the operation here proposed, to determine whether or not the pulp has been reached by the caries.

When we are satisfied, from examination, that the caries has not nearly reached the pulp, and can be removed without danger, the course is plain.

In examining a cavity of decay when there is any reason to suspect that the pulp has been reached, I generally proceed as follows: I ask the patient if he has experienced pain? If so, of what character? If there has been none of any kind, I take it for granted that the pulp is not yet actually reached by the caries, and proceed to the preparation of the cavity.

If pain has occurred, I inquire whether it was of such character as would justify the belief that it is tooth-ache proceeding from an exposed or inflamed pulp.* If any considerable pain has occurred and continued for any length of time, the preservation of the affected tooth, by the means here indicated, is very doubtful, and until some experience has been had, which will enable the operator to discriminate in these cases, it will be better to proceed at once to the extirpation of the pulp.

If, however, the pain has been inconsiderable, indicating only some slight irritation, caused by the proximity of the caries to the pulp, or from extreme inflammation of the dentine, I proceed to a close examination of the cavity. I first wash out all loose extraneous matter, with the syringe, dry it carefully in the usual way, and examine closely with the eye. I then press firmly into the cavity, with a blunt instrument, a piece of cotton or lint. If pain is produced only whilst the pressure is continued; and immediately ceases on its being removed, the indications are favorable, and I go on to the preparation of the cavity.

I first remove the carious bone from every part except that directly at the point where the pulp is most prominent. I then carefully prepare the opening of the cavity, and complete its preparation, by removing as much of the softened bone, directly over the pulp, with light touches of the instrument, as can be done with perfect safety; if there is the least danger of uncovering the pulp, I do not touch this part at all, but, without any hesitation, leave discolored and decomposed bone at the bottom of the cavity. If it will bear enough pressure, I fill with gold, firmly, and mark the case in my note book, for future examination. If there is much tenderness to pressure, I frequently cover the bottom of the cavity with a piece of gold plate, so as to relieve the most tender point, by distributing the pressure over the whole surface. In using a piece of plate for this purpose, in the upper teeth, it will be found convenient to

---

* I would here refer to the first of three articles published in the "Dental News Letter," on the treatment of the Dental Pulp, by Dr. J. D. White, of Philadelphia, in which the varieties of tooth-ache which occur are referred to their true causes.

fix it in place by warming it slightly, and touching it to a piece of gutta percha. It will take up enough, and a very slight quantity is enough to fix it in place.

Sometimes, as in the case above cited, the decay will be found so extensive as to make a temporizing course of treatment necessary. In the statement of that case, I have sufficiently indicated my mode of practice.

After a filling in such cases as these is completed, either intended for a temporary purpose, or otherwise, the tooth must be carefully watched for a time. If a decided tooth-ache should occur in a tooth so treated, it is pretty conclusive evidence that the pulp has became inflamed, and its removal will become necessary. When this is decided upon, the sooner it is done the better. If the patient should endure the pain for a few days and it should then cease, the removal of the filling and the pulp is just as imperatively called for in most cases, for the suspension of the pain is a mere delusive calm, and will, inevitably, at some future time, return in the form of a very obstinate inflammation of the peridental membrane.

It sometimes happens, and this certainly forms one of the most serious objections to the practice which I have recommended, that the pulp gradually loses its vitality, without causing any pain which the patient will notice, until peridental inflammation, which occurs sooner or later, sets in. All that can be done to guard against this evil is, to charge your patient to come to you on the occurrence of the very first symptoms of inflammation of this membrane, so that you may take advantage of the most favorable moment for treating it. After such an operation, indeed, it will be well to see your patient as often as possible, for a time, to enable you to watch the result. When the pulp dies, a change of hue of the affected tooth always takes place, and a careful examination will detect this fact long before peridental inflammation occurs. The difference between a tooth in this condition, when the pulp first dies, and one in a perfectly healthy condition is very slight, but can, at least in the teeth near the front of the mouth, be detected in a

4

strong light by their opaque appearance. I think it may be set down as a general rule, that the pulp will be found dead when the peridental membrane is considerably inflamed, and when this occurs, the removal of the filling and pulp in cases of the kind here alluded to is indicated.

At the time of writing this sheet, I have a case in my hands strongly bearing upon this matter. A young lady, for whom I have had occasion to perform a number of operations, has been in my hands for two or three weeks. I supposed I had completed all that was necessary to be done for her, and was making a final careful examination before dismissing her, when my attention was drawn to a very slight difference in color between the right superior lateral incisor and the adjacent teeth. I had only a day or two before filled this tooth on the lingual surface, where it was very slightly decayed. On questioning the patient, I found that several years before, she had suffered violent pain for a few days in this tooth, but that it passed away, and she had suffered no pain since. On examining it again closely, I found that it had lost that life-like semi-transparent hue which marks the living tooth, but it was so slight a shade darker than the rest of the teeth, that, although I had filled it, it escaped my notice. I removed my filling, cut down to the pulp cavity, between which and the cavity of decay, there was quite a thick plate of bone, and found that not a trace of vitality remained even to the extreme point of the canal of the root. I removed the remains of the decomposed pulp from the root and filled it carefully with gold.

After filling with tin-foil, or any other substance, for a temporary purpose, I allow the filling to remain about a year, sometimes longer, before proceeding to fill finally. I take occasion, however, to make several examinations of the case in the interim, and particularly direct my patient if any thing unusual occurs, if any difference, however slight, between this and the rest of the teeth is observed, to come to me at once.

I am aware how extremely meagre these practical directions are, and how loosely all this paper is put together, but very pressing engagements have prevented me from giving to it that calm and long continued consideration which the subject deserves. My only apology for giving it for publication, in its present imperfect condition is, that I promised it at this time, and was obliged to hand it in some form to the editor of this "Journal."

